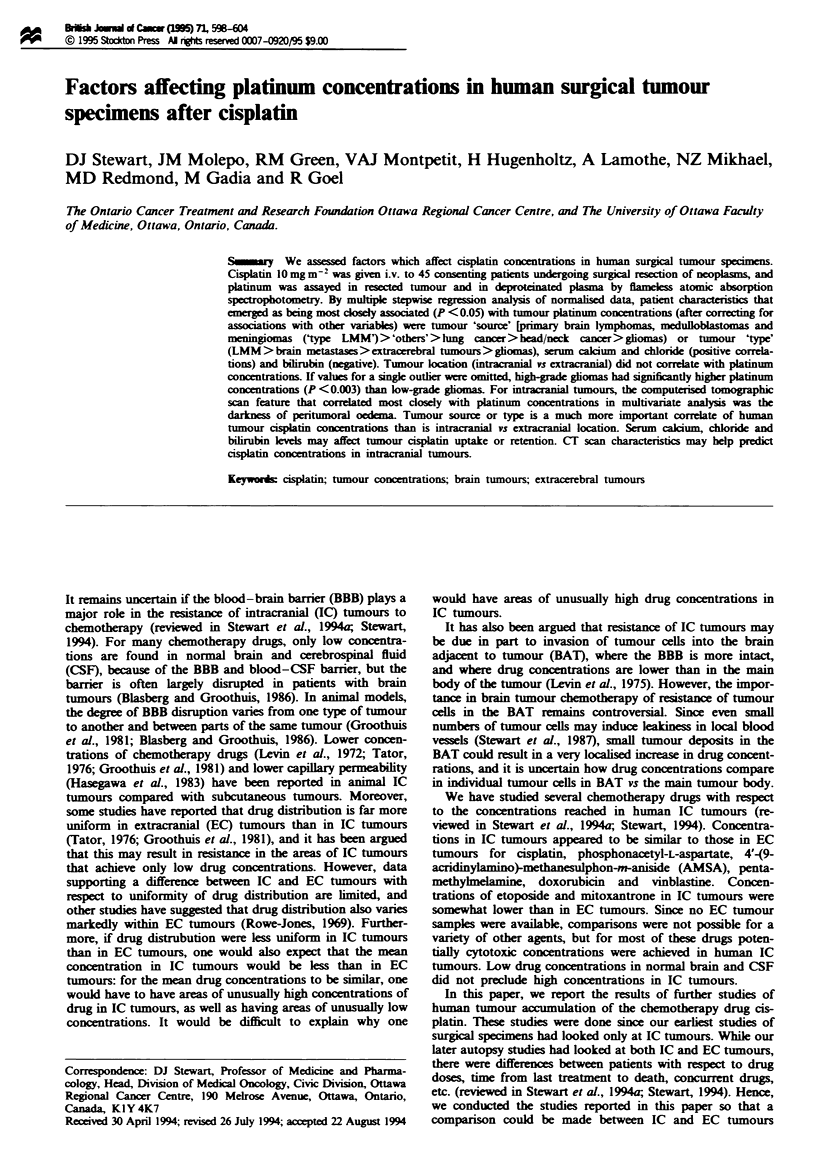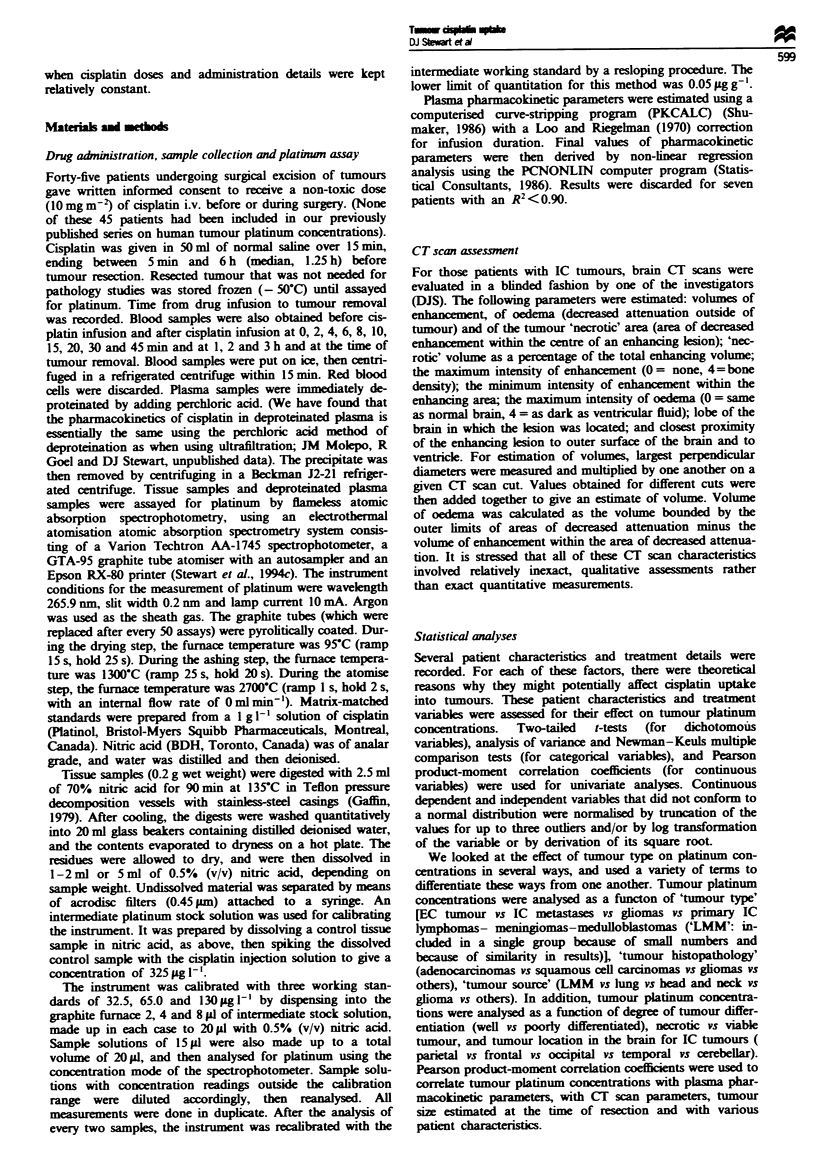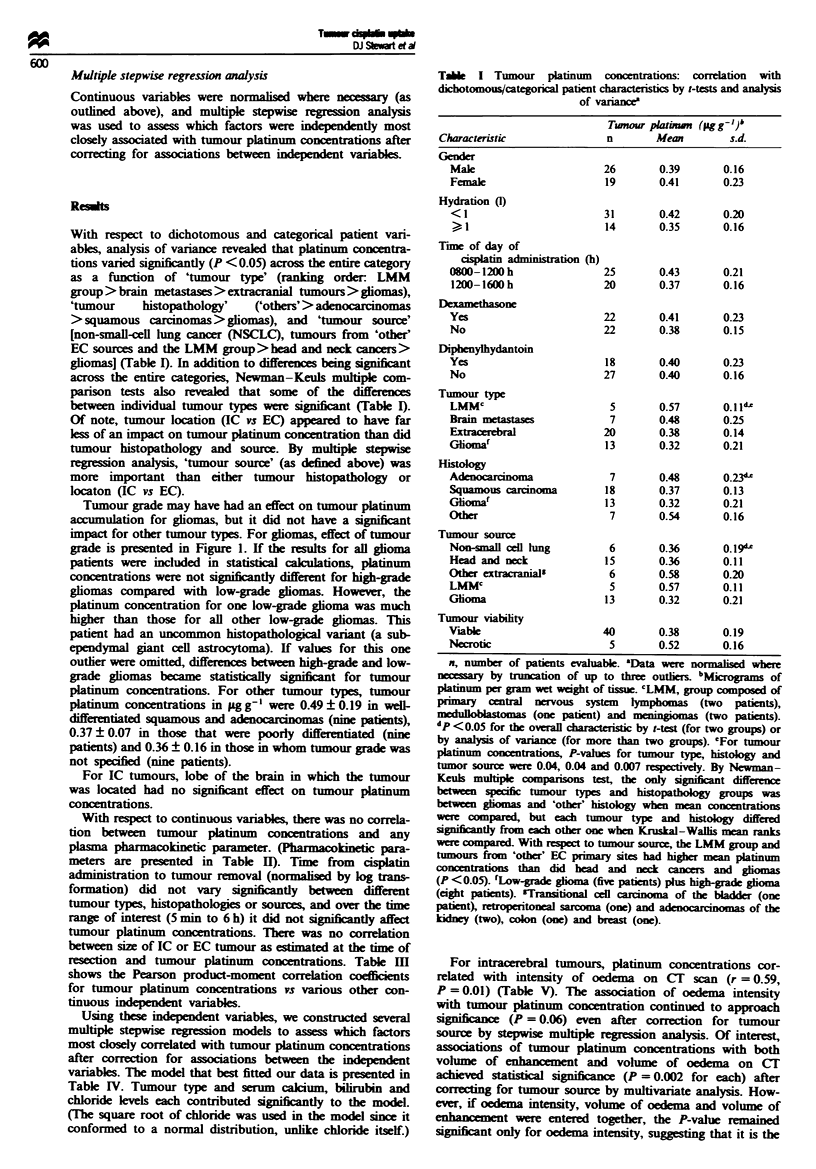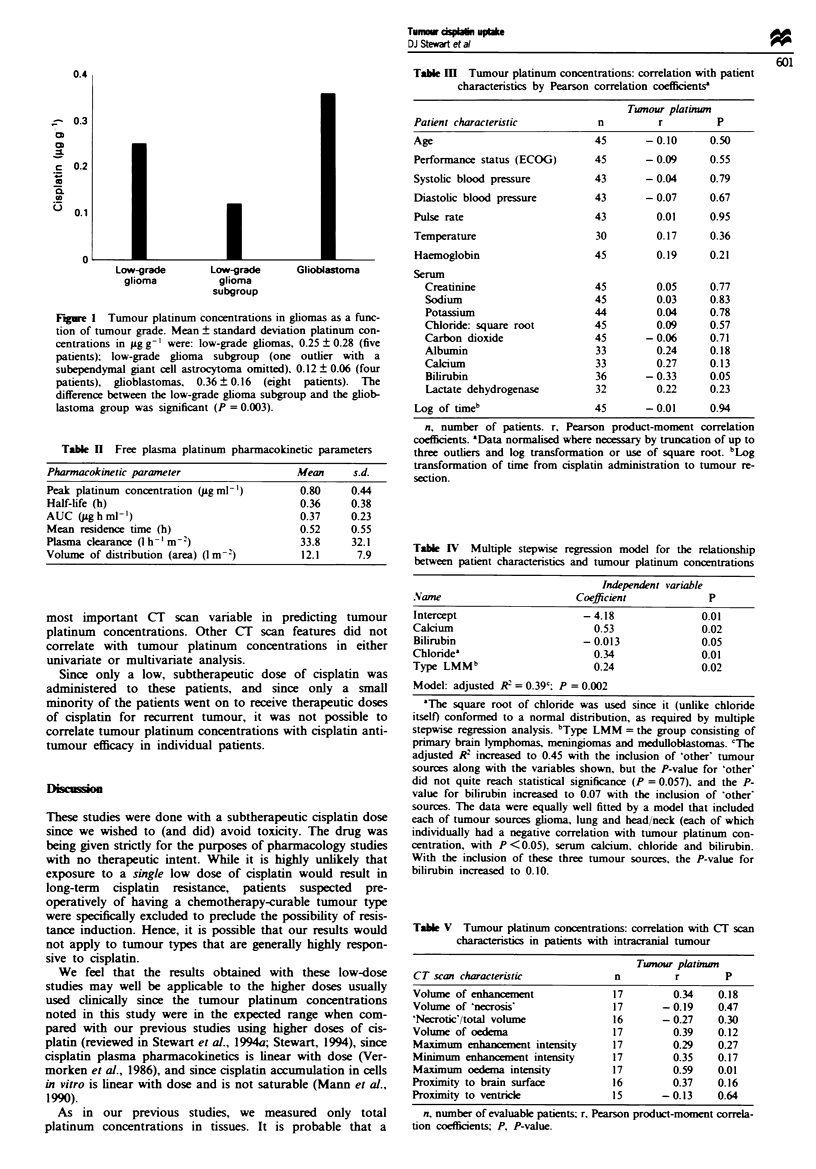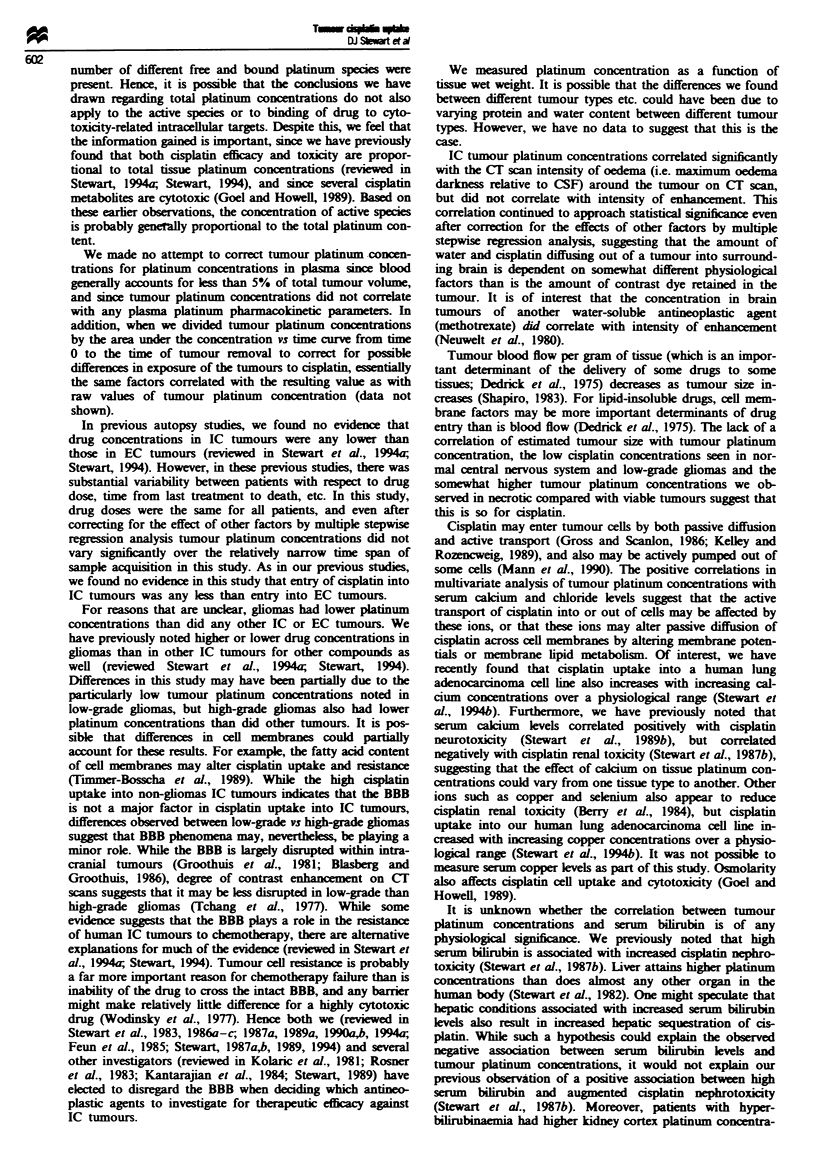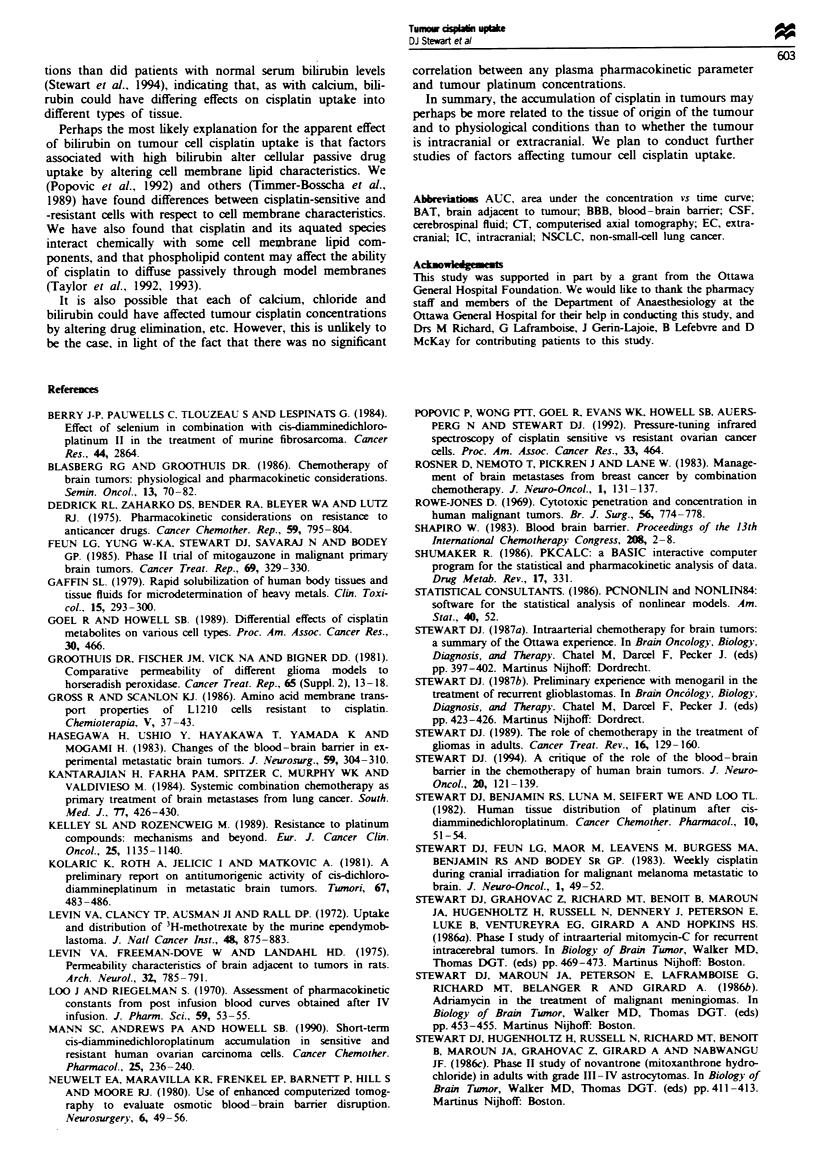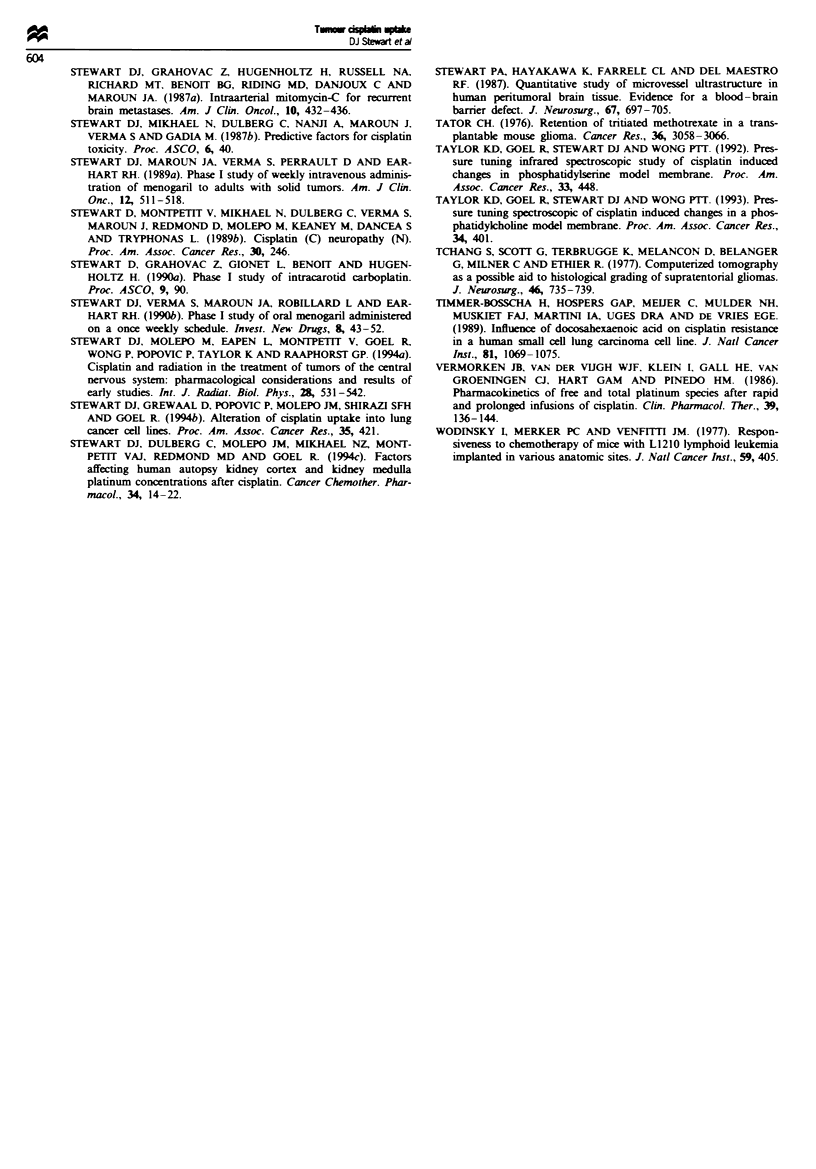# Factors affecting platinum concentrations in human surgical tumour specimens after cisplatin.

**DOI:** 10.1038/bjc.1995.116

**Published:** 1995-03

**Authors:** D. J. Stewart, J. M. Molepo, R. M. Green, V. A. Montpetit, H. Hugenholtz, A. Lamothe, N. Z. Mikhael, M. D. Redmond, M. Gadia, R. Goel

**Affiliations:** Ontario Cancer Treatment and Research Foundation, Ottawa Regional Cancer Centre, Canada.

## Abstract

We assessed factors which affect cisplatin concentrations in human surgical tumour specimens. Cisplatin 10 mg m-2 was given i.v. to 45 consenting patients undergoing surgical resection of neoplasms, and platinum was assayed in resected tumour and in deproteinated plasma by flameless atomic absorption spectrophotometry. By multiple stepwise regression analysis of normalised data, patient characteristics that emerged as being most closely associated (P < 0.05) with tumour platinum concentrations (after correcting for associations with other variables) were tumour 'source' [primary brain lymphomas, medulloblastomas and meningiomas ('type LMM') > 'others' > lung cancer > head/neck cancer > gliomas) or tumour 'type' (LMM > brain metastases > extracerebral tumours > gliomas), serum calcium and chloride (positive correlations) and bilirubin (negative). Tumour location (intracranial vs extracranial) did not correlate with platinum concentrations. If values for a single outlier were omitted, high-grade gliomas had significantly higher platinum concentrations (P < 0.003) than low-grade gliomas. For intracranial tumours, the computerised tomographic scan feature that correlated most closely with platinum concentrations in multivariate analysis was the darkness of peritumoral oedema. Tumour source or type is a much more important correlate of human tumour cisplatin concentrations than is intracranial vs extracranial location. Serum calcium, chloride and bilirubin levels may affect tumour cisplatin uptake or retention. CT scan characteristics may help predict cisplatin concentrations in intracranial tumours.


© 1995 Stockton Press All rights reserved 0007-0920/95 $9.00

### Factors affecting platinum concentrations in human surgical tumour specimens after cisplatin

DJ Stewart, JM Molepo, RM Green, VAJ Montpetit, H Hugenholtz, A Lamothe, NZ Mikhael, MD Redmond, M Gadia and R Goel

*The Ontario Cancer Treatment and Research Foundation Ottawa Regional Cancer Centre, and The University of Ottawa Faculty of Medicine, Ottawa, Ontario, Canada.*

**Summary** We assessed factors which affect cisplatin concentrations in human surgical tumour specimens. Cisplatin 10 mg $m^{-2}$ was given i.v. to 45 consenting patients undergoing surgical resection of neoplasms, and platinum was assayed in resected tumour and in deproteinated plasma by flameless atomic absorption spectrophotometry. By multiple stepwise regression analysis of normalised data, patient characteristics that emerged as being most closely associated ($P < 0.05$) with tumour platinum concentrations (after correcting for associations with other variables) were tumour 'source' [primary brain lymphomas, medulloblastomas and meningiomas ('type LMM') > 'others' > lung cancer > head/neck cancer > gliomas) or tumour 'type' (LMM > brain metastases > extracerebral tumours > gliomas), serum calcium and chloride (positive correlations) and bilirubin (negative). Tumour location (intracranial *vs* extracranial) did not correlate with platinum concentrations. If values for a single outlier were omitted, high-grade gliomas had significantly higher platinum concentrations ($P < 0.003$) than low-grade gliomas. For intracranial tumours, the computerised tomographic scan feature that correlated most closely with platinum concentrations in multivariate analysis was the darkness of peritumoral oedema. Tumour source or type is a much more important correlate of human tumour cisplatin concentrations than is intracranial *vs* extracranial location. Serum calcium, chloride and bilirubin levels may affect tumour cisplatin uptake or retention. CT scan characteristics may help predict cisplatin concentrations in intracranial tumours.

**Keywords:** cisplatin; tumour concentrations; brain tumours; extracerebral tumours

It remains uncertain if the blood-brain barrier (BBB) plays a major role in the resistance of intracranial (IC) tumours to chemotherapy (reviewed in Stewart *et al.*, 1994*a*; Stewart, 1994). For many chemotherapy drugs, only low concentrations are found in normal brain and cerebrospinal fluid (CSF), because of the BBB and blood-CSF barrier, but the barrier is often largely disrupted in patients with brain tumours (Blasberg and Groothuis, 1986). In animal models, the degree of BBB disruption varies from one type of tumour to another and between parts of the same tumour (Groothuis *et al.*, 1981; Blasberg and Groothuis, 1986). Lower concentrations of chemotherapy drugs (Levin *et al.*, 1972; Tator, 1976; Groothuis *et al.*, 1981) and lower capillary permeability (Hasegawa *et al.*, 1983) have been reported in animal IC tumours compared with subcutaneous tumours. Moreover, some studies have reported that drug distribution is far more uniform in extracranial (EC) tumours than in IC tumours (Tator, 1976; Groothuis *et al.*, 1981), and it has been argued that this may result in resistance in the areas of IC tumours that achieve only low drug concentrations. However, data supporting a difference between IC and EC tumours with respect to uniformity of drug distribution are limited, and other studies have suggested that drug distribution also varies markedly within EC tumours (Rowe-Jones, 1969). Furthermore, if drug distrubution were less uniform in IC tumours than in EC tumours, one would also expect that the mean concentration in IC tumours would be less than in EC tumours: for the mean drug concentrations to be similar, one would have to have areas of unusually high concentrations of drug in IC tumours, as well as having areas of unusually low concentrations. It would be difficult to explain why one would have areas of unusually high drug concentrations in IC tumours.

It has also been argued that resistance of IC tumours may be due in part to invasion of tumour cells into the brain adjacent to tumour (BAT), where the BBB is more intact, and where drug concentrations are lower than in the main body of the tumour (Levin *et al.*, 1975). However, the importance in brain tumour chemotherapy of resistance of tumour cells in the BAT remains controversial. Since even small numbers of tumour cells may induce leakiness in local blood vessels (Stewart *et al.*, 1987), small tumour deposits in the BAT could result in a very localised increase in drug concentrations, and it is uncertain how drug concentrations compare in individual tumour cells in BAT *vs* the main tumour body.

We have studied several chemotherapy drugs with respect to the concentrations reached in human IC tumours (reviewed in Stewart *et al.*, 1994*a*; Stewart, 1994). Concentrations in IC tumours appeared to be similar to those in EC tumours for cisplatin, phosphonacetyl-L-aspartate, 4'-(9-acridinylamino)-methanesulphon-*m*-aniside (AMSA), pentamethylmelamine, doxorubicin and vinblastine. Concentrations of etoposide and mitoxantrone in IC tumours were somewhat lower than in EC tumours. Since no EC tumour samples were available, comparisons were not possible for a variety of other agents, but for most of these drugs potentially cytotoxic concentrations were achieved in human IC tumours. Low drug concentrations in normal brain and CSF did not preclude high concentrations in IC tumours.

In this paper, we report the results of further studies of human tumour accumulation of the chemotherapy drug cisplatin. These studies were done since our earliest studies of surgical specimens had looked only at IC tumours. While our later autopsy studies had looked at both IC and EC tumours, there were differences between patients with respect to drug doses, time from last treatment to death, concurrent drugs, etc. (reviewed in Stewart *et al.*, 1994*a*; Stewart, 1994). Hence, we conducted the studies reported in this paper so that a comparison could be made between IC and EC tumours

Correspondence: DJ Stewart, Professor of Medicine and Pharmacology, Head, Division of Medical Oncology, Civic Division, Ottawa Regional Cancer Centre, 190 Melrose Avenue, Ottawa, Ontario, Canada, K1Y 4K7
Received 30 April 1994; revised 26 July 1994; accepted 22 August 1994

when cisplatin doses and administration details were kept relatively constant.

### Materials and methods

### *Drug administration, sample collection and platinum assay*

Forty-five patients undergoing surgical excision of tumours gave written informed consent to receive a non-toxic dose (10 mg $m^{-2}$) of cisplatin i.v. before or during surgery. (None of these 45 patients had been included in our previously published series on human tumour platinum concentrations). Cisplatin was given in 50 ml of normal saline over 15 min, ending between 5 min and 6 h (median, 1.25 h) before tumour resection. Resected tumour that was not needed for pathology studies was stored frozen (− 50°C) until assayed for platinum. Time from drug infusion to tumour removal was recorded. Blood samples were also obtained before cisplatin infusion and after cisplatin infusion at 0, 2, 4, 6, 8, 10, 15, 20, 30 and 45 min and at 1, 2 and 3 h and at the time of tumour removal. Blood samples were put on ice, then centrifuged in a refrigerated centrifuge within 15 min. Red blood cells were discarded. Plasma samples were immediately deproteinated by adding perchloric acid. (We have found that the pharmacokinetics of cisplatin in deproteinated plasma is essentially the same using the perchloric acid method of deproteination as when using ultrafiltration; JM Molepo, R Goel and DJ Stewart, unpublished data). The precipitate was then removed by centrifuging in a Beckman J2-21 refrigerated centrifuge. Tissue samples and deproteinated plasma samples were assayed for platinum by flameless atomic absorption spectrophotometry, using an electrothermal atomisation atomic absorption spectrometry system consisting of a Varion Techtron AA-1745 spectrophotometer, a GTA-95 graphite tube atomiser with an autosampler and an Epson RX-80 printer (Stewart *et al*., 1994*c*). The instrument conditions for the measurement of platinum were wavelength 265.9 nm, slit width 0.2 nm and lamp current 10 mA. Argon was used as the sheath gas. The graphite tubes (which were replaced after every 50 assays) were pyrolitically coated. During the drying step, the furnace temperature was 95°C (ramp 15 s, hold 25 s). During the ashing step, the furnace temperature was 1300°C (ramp 25 s, hold 20 s). During the atomise step, the furnace temperature was 2700°C (ramp 1 s, hold 2 s, with an internal flow rate of 0 ml $min^{-1}$). Matrix-matched standards were prepared from a 1 g $l^{-1}$ solution of cisplatin (Platinol, Bristol-Myers Squibb Pharmaceuticals, Montreal, Canada). Nitric acid (BDH, Toronto, Canada) was of analar grade, and water was distilled and then deionised.

Tissue samples (0.2 g wet weight) were digested with 2.5 ml of 70% nitric acid for 90 min at 135°C in Teflon pressure decomposition vessels with stainless-steel casings (Gaffin, 1979). After cooling, the digests were washed quantitatively into 20 ml glass beakers containing distilled deionised water, and the contents evaporated to dryness on a hot plate. The residues were allowed to dry, and were then dissolved in 1–2 ml or 5 ml of 0.5% (v/v) nitric acid, depending on sample weight. Undissolved material was separated by means of acrodisc filters (0.45 μm) attached to a syringe. An intermediate platinum stock solution was used for calibrating the instrument. It was prepared by dissolving a control tissue sample in nitric acid, as above, then spiking the dissolved control sample with the cisplatin injection solution to give a concentration of 325 μg $l^{-1}$.

The instrument was calibrated with three working standards of 32.5, 65.0 and 130 μg $l^{-1}$ by dispensing into the graphite furnace 2, 4 and 8 μl of intermediate stock solution, made up in each case to 20 μl with 0.5% (v/v) nitric acid. Sample solutions of 15 μl were also made up to a total volume of 20 μl, and then analysed for platinum using the concentration mode of the spectrophotometer. Sample solutions with concentration readings outside the calibration range were diluted accordingly, then reanalysed. All measurements were done in duplicate. After the analysis of every two samples, the instrument was recalibrated with the intermediate working standard by a resloping procedure. The lower limit of quantitation for this method was 0.05 μg $g^{-1}$.

Plasma pharmacokinetic parameters were estimated using a computerised curve-stripping program (PKCALC) (Shumaker, 1986) with a Loo and Riegelman (1970) correction for infusion duration. Final values of pharmacokinetic parameters were then derived by non-linear regression analysis using the PCNONLIN computer program (Statistical Consultants, 1986). Results were discarded for seven patients with an $R^2 < 0.90$.

### *CT scan assessment*

For those patients with IC tumours, brain CT scans were evaluated in a blinded fashion by one of the investigators (DJS). The following parameters were estimated: volumes of enhancement, of oedema (decreased attenuation outside of tumour) and of the tumour 'necrotic' area (area of decreased enhancement within the centre of an enhancing lesion); 'necrotic' volume as a percentage of the total enhancing volume; the maximum intensity of enhancement (0 = none, 4 = bone density); the minimum intensity of enhancement within the enhancing area; the maximum intensity of oedema (0 = same as normal brain, 4 = as dark as ventricular fluid); lobe of the brain in which the lesion was located; and closest proximity of the enhancing lesion to outer surface of the brain and to ventricle. For estimation of volumes, largest perpendicular diameters were measured and multiplied by one another on a given CT scan cut. Values obtained for different cuts were then added together to give an estimate of volume. Volume of oedema was calculated as the volume bounded by the outer limits of areas of decreased attenuation minus the volume of enhancement within the area of decreased attenuation. It is stressed that all of these CT scan characteristics involved relatively inexact, qualitative assessments rather than exact quantitative measurements.

### *Statistical analyses*

Several patient characteristics and treatment details were recorded. For each of these factors, there were theoretical reasons why they might potentially affect cisplatin uptake into tumours. These patient characteristics and treatment variables were assessed for their effect on tumour platinum concentrations. Two-tailed *t*-tests (for dichotomous variables), analysis of variance and Newman–Keuls multiple comparison tests (for categorical variables), and Pearson product-moment correlation coefficients (for continuous variables) were used for univariate analyses. Continuous dependent and independent variables that did not conform to a normal distribution were normalised by truncation of the values for up to three outliers and/or by log transformation of the variable or by derivation of its square root.

We looked at the effect of tumour type on platinum concentrations in several ways, and used a variety of terms to differentiate these ways from one another. Tumour platinum concentrations were analysed as a functon of 'tumour type' [EC tumour *vs* IC metastases *vs* gliomas *vs* primary IC lymphomas– meningiomas–medulloblastomas ('LMM': included in a single group because of small numbers and because of similarity in results)], 'tumour histopathology' (adenocarcinomas *vs* squamous cell carcinomas *vs* gliomas *vs* others), 'tumour source' (LMM *vs* lung *vs* head and neck *vs* glioma *vs* others). In addition, tumour platinum concentrations were analysed as a function of degree of tumour differentiation (well *vs* poorly differentiated), necrotic *vs* viable tumour, and tumour location in the brain for IC tumours ( parietal *vs* frontal *vs* occipital *vs* temporal *vs* cerebellar). Pearson product-moment correlation coefficients were used to correlate tumour platinum concentrations with plasma pharmacokinetic parameters, with CT scan parameters, tumour size estimated at the time of resection and with various patient characteristics.

*Multiple stepwise regression analysis*

Continuous variables were normalised where necessary (as outlined above), and multiple stepwise regression analysis was used to assess which factors were independently most closely associated with tumour platinum concentrations after correcting for associations between independent variables.

### Results

With respect to dichotomous and categorical patient variables, analysis of variance revealed that platinum concentrations varied significantly ($P < 0.05$) across the entire category as a function of 'tumour type' (ranking order: LMM group > brain metastases > extracranial tumours > gliomas), 'tumour histopathology' ('others' > adenocarcinomas > squamous carcinomas > gliomas), and 'tumour source' [non-small-cell lung cancer (NSCLC), tumours from 'other' EC sources and the LMM group > head and neck cancers > gliomas] (Table I). In addition to differences being significant across the entire categories, Newman–Keuls multiple comparison tests also revealed that some of the differences between individual tumour types were significant (Table I). Of note, tumour location (IC *vs* EC) appeared to have far less of an impact on tumour platinum concentration than did tumour histopathology and source. By multiple stepwise regression analysis, 'tumour source' (as defined above) was more important than either tumour histopathology or locaton (IC *vs* EC).

Tumour grade may have had an effect on tumour platinum accumulation for gliomas, but it did not have a significant impact for other tumour types. For gliomas, effect of tumour grade is presented in Figure 1. If the results for all glioma patients were included in statistical calculations, platinum concentrations were not significantly different for high-grade gliomas compared with low-grade gliomas. However, the platinum concentration for one low-grade glioma was much higher than those for all other low-grade gliomas. This patient had an uncommon histopathological variant (a subependymal giant cell astrocytoma). If values for this one outlier were omitted, differences between high-grade and low-grade gliomas became statistically significant for tumour platinum concentrations. For other tumour types, tumour platinum concentrations in $\mu g\ g^{-1}$ were $0.49 \pm 0.19$ in well-differentiated squamous and adenocarcinomas (nine patients), $0.37 \pm 0.07$ in those that were poorly differentiated (nine patients) and $0.36 \pm 0.16$ in those in whom tumour grade was not specified (nine patients).

For IC tumours, lobe of the brain in which the tumour was located had no significant effect on tumour platinum concentrations.

With respect to continuous variables, there was no correlation between tumour platinum concentrations and any plasma pharmacokinetic parameter. (Pharmacokinetic parameters are presented in Table II). Time from cisplatin administration to tumour removal (normalised by log transformation) did not vary significantly between different tumour types, histopathologies or sources, and over the time range of interest (5 min to 6 h) it did not significantly affect tumour platinum concentrations. There was no correlation between size of IC or EC tumour as estimated at the time of resection and tumour platinum concentrations. Table III shows the Pearson product-moment correlation coefficients for tumour platinum concentrations *vs* various other continuous independent variables.

Using these independent variables, we constructed several multiple stepwise regression models to assess which factors most closely correlated with tumour platinum concentrations after correction for associations between the independent variables. The model that best fitted our data is presented in Table IV. Tumour type and serum calcium, bilirubin and chloride levels each contributed significantly to the model. (The square root of chloride was used in the model since it conformed to a normal distribution, unlike chloride itself.)

**Table I** Tumour platinum concentrations: correlation with dichotomous/categorical patient characteristics by *t*-tests and analysis of variance[a]

| *Characteristic* | n | *Tumour platinum* ($\mu g\ g^{-1}$)[b] *Mean* | *s.d.* |
|---|---|---|---|
| Gender | | | |
| Male | 26 | 0.39 | 0.16 |
| Female | 19 | 0.41 | 0.23 |
| Hydration (l) | | | |
| <1 | 31 | 0.42 | 0.20 |
| ⩾1 | 14 | 0.35 | 0.16 |
| Time of day of cisplatin administration (h) | | | |
| 0800–1200 h | 25 | 0.43 | 0.21 |
| 1200–1600 h | 20 | 0.37 | 0.16 |
| Dexamethasone | | | |
| Yes | 22 | 0.41 | 0.23 |
| No | 22 | 0.38 | 0.15 |
| Diphenylhydantoin | | | |
| Yes | 18 | 0.40 | 0.23 |
| No | 27 | 0.40 | 0.16 |
| Tumour type | | | |
| LMM[c] | 5 | 0.57 | 0.11[d,e] |
| Brain metastases | 7 | 0.48 | 0.25 |
| Extracerebral | 20 | 0.38 | 0.14 |
| Glioma[f] | 13 | 0.32 | 0.21 |
| Histology | | | |
| Adenocarcinoma | 7 | 0.48 | 0.23[d,e] |
| Squamous carcinoma | 18 | 0.37 | 0.13 |
| Glioma[f] | 13 | 0.32 | 0.21 |
| Other | 7 | 0.54 | 0.16 |
| Tumour source | | | |
| Non-small cell lung | 6 | 0.36 | 0.19[d,e] |
| Head and neck | 15 | 0.36 | 0.11 |
| Other extracranial[g] | 6 | 0.58 | 0.20 |
| LMM[c] | 5 | 0.57 | 0.11 |
| Glioma | 13 | 0.32 | 0.21 |
| Tumour viability | | | |
| Viable | 40 | 0.38 | 0.19 |
| Necrotic | 5 | 0.52 | 0.16 |

*n*, number of patients evaluable. [a]Data were normalised where necessary by truncation of up to three outliers. [b]Micrograms of platinum per gram wet weight of tissue. [c]LMM, group composed of primary central nervous system lymphomas (two patients), medulloblastomas (one patient) and meningiomas (two patients). [d]$P < 0.05$ for the overall characteristic by *t*-test (for two groups) or by analysis of variance (for more than two groups). [e]For tumour platinum concentrations, *P*-values for tumour type, histology and tumor source were 0.04, 0.04 and 0.007 respectively. By Newman–Keuls multiple comparisons test, the only significant difference between specific tumour types and histopathology groups was between gliomas and 'other' histology when mean concentrations were compared, but each tumour type and histology differed significantly from each other one when Kruskal–Wallis mean ranks were compared. With respect to tumour source, the LMM group and tumours from 'other' EC primary sites had higher mean platinum concentrations than did head and neck cancers and gliomas ($P < 0.05$). [f]Low-grade glioma (five patients) plus high-grade glioma (eight patients). [g]Transitional cell carcinoma of the bladder (one patient), retroperitoneal sarcoma (one) and adenocarcinomas of the kidney (two), colon (one) and breast (one).

For intracerebral tumours, platinum concentrations correlated with intensity of oedema on CT scan ($r = 0.59$, $P = 0.01$) (Table V). The association of oedema intensity with tumour platinum concentration continued to approach significance ($P = 0.06$) even after correction for tumour source by stepwise multiple regression analysis. Of interest, associations of tumour platinum concentrations with both volume of enhancement and volume of oedema on CT achieved statistical significance ($P = 0.002$ for each) after correcting for tumour source by multivariate analysis. However, if oedema intensity, volume of oedema and volume of enhancement were entered together, the *P*-value remained significant only for oedema intensity, suggesting that it is the

**Figure 1** Tumour platinum concentrations in gliomas as a function of tumour grade. Mean ± standard deviation platinum concentrations in $\mu g\ g^{-1}$ were: low-grade gliomas, 0.25 ± 0.28 (five patients); low-grade glioma subgroup (one outlier with a subependymal giant cell astrocytoma omitted), 0.12 ± 0.06 (four patients), glioblastomas, 0.36 ± 0.16 (eight patients). The difference between the low-grade glioma subgroup and the glioblastoma group was significant ($P = 0.003$).

**Table II** Free plasma platinum pharmacokinetic parameters

| *Pharmacokinetic parameter* | *Mean* | *s.d.* |
|---|---|---|
| Peak platinum concentration ($\mu g\ ml^{-1}$) | 0.80 | 0.44 |
| Half-life (h) | 0.36 | 0.38 |
| AUC ($\mu g\ h\ ml^{-1}$) | 0.37 | 0.23 |
| Mean residence time (h) | 0.52 | 0.55 |
| Plasma clearance ($l\ h^{-1}\ m^{-2}$) | 33.8 | 32.1 |
| Volume of distribution (area) ($l\ m^{-2}$) | 12.1 | 7.9 |

most important CT scan variable in predicting tumour platinum concentrations. Other CT scan features did not correlate with tumour platinum concentrations in either univariate or multivariate analysis.

Since only a low, subtherapeutic dose of cisplatin was administered to these patients, and since only a small minority of the patients went on to receive therapeutic doses of cisplatin for recurrent tumour, it was not possible to correlate tumour platinum concentrations with cisplatin antitumour efficacy in individual patients.

### Discussion

These studies were done with a subtherapeutic cisplatin dose since we wished to (and did) avoid toxicity. The drug was being given strictly for the purposes of pharmacology studies with no therapeutic intent. While it is highly unlikely that exposure to a *single* low dose of cisplatin would result in long-term cisplatin resistance, patients suspected preoperatively of having a chemotherapy-curable tumour type were specifically excluded to preclude the possibility of resistance induction. Hence, it is possible that our results would not apply to tumour types that are generally highly responsive to cisplatin.

We feel that the results obtained with these low-dose studies may well be applicable to the higher doses usually used clinically since the tumour platinum concentrations noted in this study were in the expected range when compared with our previous studies using higher doses of cisplatin (reviewed in Stewart *et al.*, 1994*a*; Stewart, 1994), since cisplatin plasma pharmacokinetics is linear with dose (Vermorken *et al.*, 1986), and since cisplatin accumulation in cells *in vitro* is linear with dose and is not saturable (Mann *et al.*, 1990).

As in our previous studies, we measured only total platinum concentrations in tissues. It is probable that a

**Table III** Tumour platinum concentrations: correlation with patient characteristics by Pearson correlation coefficients[a]

| *Patient characteristic* | n | *Tumour platinum* r | P |
|---|---|---|---|
| Age | 45 | −0.10 | 0.50 |
| Performance status (ECOG) | 45 | −0.09 | 0.55 |
| Systolic blood pressure | 43 | −0.04 | 0.79 |
| Diastolic blood pressure | 43 | −0.07 | 0.67 |
| Pulse rate | 43 | 0.01 | 0.95 |
| Temperature | 30 | 0.17 | 0.36 |
| Haemoglobin | 45 | 0.19 | 0.21 |
| Serum | | | |
| Creatinine | 45 | 0.05 | 0.77 |
| Sodium | 45 | 0.03 | 0.83 |
| Potassium | 44 | 0.04 | 0.78 |
| Chloride: square root | 45 | 0.09 | 0.57 |
| Carbon dioxide | 45 | −0.06 | 0.71 |
| Albumin | 33 | 0.24 | 0.18 |
| Calcium | 33 | 0.27 | 0.13 |
| Bilirubin | 36 | −0.33 | 0.05 |
| Lactate dehydrogenase | 32 | 0.22 | 0.23 |
| Log of time[b] | 45 | −0.01 | 0.94 |

*n*, number of patients. r, Pearson product-moment correlation coefficients. [a]Data normalised where necessary by truncation of up to three outliers and log transformation or use of square root. [b]Log transformation of time from cisplatin administration to tumour resection.

**Table IV** Multiple stepwise regression model for the relationship between patient characteristics and tumour platinum concentrations

| *Name* | *Independent variable Coefficient* | P |
|---|---|---|
| Intercept | −4.18 | 0.01 |
| Calcium | 0.53 | 0.02 |
| Bilirubin | −0.013 | 0.05 |
| Chloride[a] | 0.34 | 0.01 |
| Type LMM[b] | 0.24 | 0.02 |
| Model: adjusted $R^2 = 0.39^c$; $P = 0.002$ | | |

[a]The square root of chloride was used since it (unlike chloride itself) conformed to a normal distribution, as required by multiple stepwise regression analysis. [b]Type LMM = the group consisting of primary brain lymphomas, meningiomas and medulloblastomas. [c]The adjusted $R^2$ increased to 0.45 with the inclusion of 'other' tumour sources along with the variables shown, but the *P*-value for 'other' did not quite reach statistical significance ($P = 0.057$), and the *P*-value for bilirubin increased to 0.07 with the inclusion of 'other' sources. The data were equally well fitted by a model that included each of tumour sources glioma, lung and head/neck (each of which individually had a negative correlation with tumour platinum concentration, with $P < 0.05$), serum calcium, chloride and bilirubin. With the inclusion of these three tumour sources, the *P*-value for bilirubin increased to 0.10.

**Table V** Tumour platinum concentrations: correlation with CT scan characteristics in patients with intracranial tumour

| *CT scan characteristic* | n | *Tumour platinum* r | P |
|---|---|---|---|
| Volume of enhancement | 17 | 0.34 | 0.18 |
| Volume of 'necrosis' | 17 | −0.19 | 0.47 |
| 'Necrotic'/total volume | 16 | −0.27 | 0.30 |
| Volume of oedema | 17 | 0.39 | 0.12 |
| Maximum enhancement intensity | 17 | 0.29 | 0.27 |
| Minimum enhancement intensity | 17 | 0.35 | 0.17 |
| Maximum oedema intensity | 17 | 0.59 | 0.01 |
| Proximity to brain surface | 16 | 0.37 | 0.16 |
| Proximity to ventricle | 15 | −0.13 | 0.64 |

*n*, number of evaluable patients; r, Pearson product-moment correlation coefficients; *P*, *P*-value.

number of different free and bound platinum species were present. Hence, it is possible that the conclusions we have drawn regarding total platinum concentrations do not also apply to the active species or to binding of drug to cytotoxicity-related intracellular targets. Despite this, we feel that the information gained is important, since we have previously found that both cisplatin efficacy and toxicity are proportional to total tissue platinum concentrations (reviewed in Stewart, 1994*a*; Stewart, 1994), and since several cisplatin metabolites are cytotoxic (Goel and Howell, 1989). Based on these earlier observations, the concentration of active species is probably generally proportional to the total platinum content.

We made no attempt to correct tumour platinum concentrations for platinum concentrations in plasma since blood generally accounts for less than 5% of total tumour volume, and since tumour platinum concentrations did not correlate with any plasma platinum pharmacokinetic parameters. In addition, when we divided tumour platinum concentrations by the area under the concentration *vs* time curve from time 0 to the time of tumour removal to correct for possible differences in exposure of the tumours to cisplatin, essentially the same factors correlated with the resulting value as with raw values of tumour platinum concentration (data not shown).

In previous autopsy studies, we found no evidence that drug concentrations in IC tumours were any lower than those in EC tumours (reviewed in Stewart *et al.*, 1994*a*; Stewart, 1994). However, in these previous studies, there was substantial variability between patients with respect to drug dose, time from last treatment to death, etc. In this study, drug doses were the same for all patients, and even after correcting for the effect of other factors by multiple stepwise regression analysis tumour platinum concentrations did not vary significantly over the relatively narrow time span of sample acquisition in this study. As in our previous studies, we found no evidence in this study that entry of cisplatin into IC tumours was any less than entry into EC tumours.

For reasons that are unclear, gliomas had lower platinum concentrations than did any other IC or EC tumours. We have previously noted higher or lower drug concentrations in gliomas than in other IC tumours for other compounds as well (reviewed Stewart *et al.*, 1994*a*; Stewart, 1994). Differences in this study may have been partially due to the particularly low tumour platinum concentrations noted in low-grade gliomas, but high-grade gliomas also had lower platinum concentrations than did other tumours. It is possible that differences in cell membranes could partially account for these results. For example, the fatty acid content of cell membranes may alter cisplatin uptake and resistance (Timmer-Bosscha *et al.*, 1989). While the high cisplatin uptake into non-gliomas IC tumours indicates that the BBB is not a major factor in cisplatin uptake into IC tumours, differences observed between low-grade *vs* high-grade gliomas suggest that BBB phenomena may, nevertheless, be playing a minor role. While the BBB is largely disrupted within intracranial tumours (Groothuis *et al.*, 1981; Blasberg and Groothuis, 1986), degree of contrast enhancement on CT scans suggests that it may be less disrupted in low-grade than high-grade gliomas (Tchang *et al.*, 1977). While some evidence suggests that the BBB plays a role in the resistance of human IC tumours to chemotherapy, there are alternative explanations for much of the evidence (reviewed in Stewart *et al.*, 1994*a*; Stewart, 1994). Tumour cell resistance is probably a far more important reason for chemotherapy failure than is inability of the drug to cross the intact BBB, and any barrier might make relatively little difference for a highly cytotoxic drug (Wodinsky *et al.*, 1977). Hence both we (reviewed in Stewart *et al.*, 1983, 1986*a–c*; 1987*a*, 1989*a*, 1990*a,b*, 1994*a*; Feun *et al.*, 1985; Stewart, 1987*a,b*, 1989, 1994) and several other investigators (reviewed in Kolaric *et al.*, 1981; Rosner *et al.*, 1983; Kantarajian *et al.*, 1984; Stewart, 1989) have elected to disregard the BBB when deciding which antineoplastic agents to investigate for therapeutic efficacy against IC tumours.

We measured platinum concentration as a function of tissue wet weight. It is possible that the differences we found between different tumour types etc. could have been due to varying protein and water content between different tumour types. However, we have no data to suggest that this is the case.

IC tumour platinum concentrations correlated significantly with the CT scan intensity of oedema (i.e. maximum oedema darkness relative to CSF) around the tumour on CT scan, but did not correlate with intensity of enhancement. This correlation continued to approach statistical significance even after correction for the effects of other factors by multiple stepwise regression analysis, suggesting that the amount of water and cisplatin diffusing out of a tumour into surrounding brain is dependent on somewhat different physiological factors than is the amount of contrast dye retained in the tumour. It is of interest that the concentration in brain tumours of another water-soluble antineoplastic agent (methotrexate) *did* correlate with intensity of enhancement (Neuwelt *et al.*, 1980).

Tumour blood flow per gram of tissue (which is an important determinant of the delivery of some drugs to some tissues; Dedrick *et al.*, 1975) decreases as tumour size increases (Shapiro, 1983). For lipid-insoluble drugs, cell membrane factors may be more important determinants of drug entry than is blood flow (Dedrick *et al.*, 1975). The lack of a correlation of estimated tumour size with tumour platinum concentration, the low cisplatin concentrations seen in normal central nervous system and low-grade gliomas and the somewhat higher tumour platinum concentrations we observed in necrotic compared with viable tumours suggest that this is so for cisplatin.

Cisplatin may enter tumour cells by both passive diffusion and active transport (Gross and Scanlon, 1986; Kelley and Rozencweig, 1989), and also may be actively pumped out of some cells (Mann *et al.*, 1990). The positive correlations in multivariate analysis of tumour platinum concentrations with serum calcium and chloride levels suggest that the active transport of cisplatin into or out of cells may be affected by these ions, or that these ions may alter passive diffusion of cisplatin across cell membranes by altering membrane potentials or membrane lipid metabolism. Of interest, we have recently found that cisplatin uptake into a human lung adenocarcinoma cell line also increases with increasing calcium concentrations over a physiological range (Stewart *et al.*, 1994*b*). Furthermore, we have previously noted that serum calcium levels correlated positively with cisplatin neurotoxicity (Stewart *et al.*, 1989*b*), but correlated negatively with cisplatin renal toxicity (Stewart *et al.*, 1987*b*), suggesting that the effect of calcium on tissue platinum concentrations could vary from one tissue type to another. Other ions such as copper and selenium also appear to reduce cisplatin renal toxicity (Berry *et al.*, 1984), but cisplatin uptake into our human lung adenocarcinoma cell line increased with increasing copper concentrations over a physiological range (Stewart *et al.*, 1994*b*). It was not possible to measure serum copper levels as part of this study. Osmolarity also affects cisplatin cell uptake and cytotoxicity (Goel and Howell, 1989).

It is unknown whether the correlation between tumour platinum concentrations and serum bilirubin is of any physiological significance. We previously noted that high serum bilirubin is associated with increased cisplatin nephrotoxicity (Stewart *et al.*, 1987*b*). Liver attains higher platinum concentrations than does almost any other organ in the human body (Stewart *et al.*, 1982). One might speculate that hepatic conditions associated with increased serum bilirubin levels also result in increased hepatic sequestration of cisplatin. While such a hypothesis could explain the observed negative association between serum bilirubin levels and tumour platinum concentrations, it would not explain our previous observation of a positive association between high serum bilirubin and augmented cisplatin nephrotoxicity (Stewart *et al.*, 1987*b*). Moreover, patients with hyperbilirubinaemia had higher kidney cortex platinum concentrations than did patients with normal serum bilirubin levels (Stewart *et al.*, 1994), indicating that, as with calcium, bilirubin could have differing effects on cisplatin uptake into different types of tissue.

Perhaps the most likely explanation for the apparent effect of bilirubin on tumour cell cisplatin uptake is that factors associated with high bilirubin alter cellular passive drug uptake by altering cell membrane lipid characteristics. We (Popovic *et al.*, 1992) and others (Timmer-Bosscha *et al.*, 1989) have found differences between cisplatin-sensitive and -resistant cells with respect to cell membrane characteristics. We have also found that cisplatin and its aquated species interact chemically with some cell membrane lipid components, and that phospholipid content may affect the ability of cisplatin to diffuse passively through model membranes (Taylor *et al.*, 1992, 1993).

It is also possible that each of calcium, chloride and bilirubin could have affected tumour cisplatin concentrations by altering drug elimination, etc. However, this is unlikely to be the case, in light of the fact that there was no significant correlation between any plasma pharmacokinetic parameter and tumour platinum concentrations.

In summary, the accumulation of cisplatin in tumours may perhaps be more related to the tissue of origin of the tumour and to physiological conditions than to whether the tumour is intracranial or extracranial. We plan to conduct further studies of factors affecting tumour cell cisplatin uptake.

**Abbreviations** AUC, area under the concentration *vs* time curve; BAT, brain adjacent to tumour; BBB, blood–brain barrier; CSF, cerebrospinal fluid; CT, computerised axial tomography; EC, extracranial; IC, intracranial; NSCLC, non-small-cell lung cancer.

**Acknowledgements**
This study was supported in part by a grant from the Ottawa General Hospital Foundation. We would like to thank the pharmacy staff and members of the Department of Anaesthesiology at the Ottawa General Hospital for their help in conducting this study, and Drs M Richard, G Laframboise, J Gerin-Lajoie, B Lefebvre and D McKay for contributing patients to this study.

**References**

BERRY J-P, PAUWELLS C, TLOUZEAU S AND LESPINATS G. (1984). Effect of selenium in combination with cis-diamminedichloroplatinum II in the treatment of murine fibrosarcoma. *Cancer Res.*, **44**, 2864.

BLASBERG RG AND GROOTHUIS DR. (1986). Chemotherapy of brain tumors: physiological and pharmacokinetic considerations. *Semin. Oncol.*, **13**, 70–82.

DEDRICK RL, ZAHARKO DS, BENDER RA, BLEYER WA AND LUTZ RJ. (1975). Pharmacokinetic considerations on resistance to anticancer drugs. *Cancer Chemother. Rep.*, **59**, 795–804.

FEUN LG, YUNG W-KA, STEWART DJ, SAVARAJ N AND BODEY GP. (1985). Phase II trial of mitogauzone in malignant primary brain tumors. *Cancer Treat. Rep.*, **69**, 329–330.

GAFFIN SL. (1979). Rapid solubilization of human body tissues and tissue fluids for microdetermination of heavy metals. *Clin. Toxicol.*, **15**, 293–300.

GOEL R AND HOWELL SB. (1989). Differential effects of cisplatin metabolites on various cell types. *Proc. Am. Assoc. Cancer Res.*, **30**, 466.

GROOTHUIS DR, FISCHER JM, VICK NA AND BIGNER DD. (1981). Comparative permeability of different glioma models to horseradish peroxidase. *Cancer Treat. Rep.*, **65** (Suppl. 2), 13–18.

GROSS R AND SCANLON KJ. (1986). Amino acid membrane transport properties of L1210 cells resistant to cisplatin. *Chemioterapia*, **V**, 37–43.

HASEGAWA H, USHIO Y, HAYAKAWA T, YAMADA K AND MOGAMI H. (1983). Changes of the blood–brain barrier in experimental metastatic brain tumors. *J. Neurosurg.*, **59**, 304–310.

KANTARAJIAN H, FARHA PAM, SPITZER C, MURPHY WK AND VALDIVIESO M. (1984). Systemic combination chemotherapy as primary treatment of brain metastases from lung cancer. *South. Med. J.*, **77**, 426–430.

KELLEY SL AND ROZENCWEIG M. (1989). Resistance to platinum compounds: mechanisms and beyond. *Eur. J. Cancer Clin. Oncol.*, **25**, 1135–1140.

KOLARIC K, ROTH A, JELICIC I AND MATKOVIC A. (1981). A preliminary report on antitumorigenic activity of cis-dichlorodiammineplatinum in metastatic brain tumors. *Tumori*, **67**, 483–486.

LEVIN VA, CLANCY TP, AUSMAN JI AND RALL DP. (1972). Uptake and distribution of $^3$H-methotrexate by the murine ependymoblastoma. *J. Natl Cancer Inst.*, **48**, 875–883.

LEVIN VA, FREEMAN-DOVE W AND LANDAHL HD. (1975). Permeability characteristics of brain adjacent to tumors in rats. *Arch. Neurol.*, **32**, 785–791.

LOO J AND RIEGELMAN S. (1970). Assessment of pharmacokinetic constants from post infusion blood curves obtained after IV infusion. *J. Pharm. Sci.*, **59**, 53–55.

MANN SC, ANDREWS PA AND HOWELL SB. (1990). Short-term cis-diamminedichloroplatinum accumulation in sensitive and resistant human ovarian carcinoma cells. *Cancer Chemother. Pharmacol.*, **25**, 236–240.

NEUWELT EA, MARAVILLA KR, FRENKEL EP, BARNETT P, HILL S AND MOORE RJ. (1980). Use of enhanced computerized tomography to evaluate osmotic blood–brain barrier disruption. *Neurosurgery*, **6**, 49–56.

POPOVIC P, WONG PTT, GOEL R, EVANS WK, HOWELL SB, AUERSPERG N AND STEWART DJ. (1992). Pressure-tuning infrared spectroscopy of cisplatin sensitive vs resistant ovarian cancer cells. *Proc. Am. Assoc. Cancer Res.*, **33**, 464.

ROSNER D, NEMOTO T, PICKREN J AND LANE W. (1983). Management of brain metastases from breast cancer by combination chemotherapy. *J. Neuro-Oncol.*, **1**, 131–137.

ROWE-JONES D. (1969). Cytotoxic penetration and concentration in human malignant tumors. *Br. J. Surg.*, **56**, 774–778.

SHAPIRO W. (1983). Blood brain barrier. *Proceedings of the 13th International Chemotherapy Congress*, **208**, 2–8.

SHUMAKER R. (1986). PKCALC: a BASIC interactive computer program for the statistical and pharmacokinetic analysis of data. *Drug Metab. Rev.*, **17**, 331.

STATISTICAL CONSULTANTS. (1986). PCNONLIN and NONLIN84: software for the statistical analysis of nonlinear models. *Am. Stat.*, **40**, 52.

STEWART DJ. (1987*a*). Intraarterial chemotherapy for brain tumors: a summary of the Ottawa experience. In *Brain Oncology, Biology, Diagnosis, and Therapy*. Chatel M, Darcel F, Pecker J. (eds) pp. 397–402. Martinus Nijhoff: Dordrecht.

STEWART DJ. (1987*b*). Preliminary experience with menogaril in the treatment of recurrent glioblastomas. In *Brain Oncology, Biology, Diagnosis, and Therapy*. Chatel M, Darcel F, Pecker J. (eds) pp. 423–426. Martinus Nijhoff: Dordrect.

STEWART DJ. (1989). The role of chemotherapy in the treatment of gliomas in adults. *Cancer Treat. Rev.*, **16**, 129–160.

STEWART DJ. (1994). A critique of the role of the blood–brain barrier in the chemotherapy of human brain tumors. *J. Neuro-Oncol.*, **20**, 121–139.

STEWART DJ, BENJAMIN RS, LUNA M, SEIFERT WE AND LOO TL. (1982). Human tissue distribution of platinum after cis-diamminedichloroplatinum. *Cancer Chemother. Pharmacol.*, **10**, 51–54.

STEWART DJ, FEUN LG, MAOR M, LEAVENS M, BURGESS MA, BENJAMIN RS AND BODEY SR GP. (1983). Weekly cisplatin during cranial irradiation for malignant melanoma metastatic to brain. *J. Neuro-Oncol.*, **1**, 49–52.

STEWART DJ, GRAHOVAC Z, RICHARD MT, BENOIT B, MAROUN JA, HUGENHOLTZ H, RUSSELL N, DENNERY J, PETERSON E, LUKE B, VENTUREYRA EG, GIRARD A AND HOPKINS HS. (1986*a*). Phase I study of intraarterial mitomycin-C for recurrent intracerebral tumors. In *Biology of Brain Tumor*, Walker MD, Thomas DGT. (eds) pp. 469–473. Martinus Nijhoff: Boston.

STEWART DJ, MAROUN JA, PETERSON E, LAFRAMBOISE G, RICHARD MT, BELANGER R AND GIRARD A. (1986*b*). Adriamycin in the treatment of malignant meningiomas. In *Biology of Brain Tumor*, Walker MD, Thomas DGT. (eds) pp. 453–455. Martinus Nijhoff: Boston.

STEWART DJ, HUGENHOLTZ H, RUSSELL N, RICHARD MT, BENOIT B, MAROUN JA, GRAHOVAC Z, GIRARD A AND NABWANGU JF. (1986*c*). Phase II study of novantrone (mitoxanthrone hydrochloride) in adults with grade III–IV astrocytomas. In *Biology of Brain Tumor*, Walker MD, Thomas DGT. (eds) pp. 411–413. Martinus Nijhoff: Boston.

STEWART DJ, GRAHOVAC Z, HUGENHOLTZ H, RUSSELL NA, RICHARD MT, BENOIT BG, RIDING MD, DANJOUX C AND MAROUN JA. (1987*a*). Intraarterial mitomycin-C for recurrent brain metastases. *Am. J Clin. Oncol.*, **10,** 432–436.

STEWART DJ, MIKHAEL N, DULBERG C, NANJI A, MAROUN J, VERMA S AND GADIA M. (1987*b*). Predictive factors for cisplatin toxicity. *Proc. ASCO*, **6,** 40.

STEWART DJ, MAROUN JA, VERMA S, PERRAULT D AND EARHART RH. (1989*a*). Phase I study of weekly intravenous administration of menogaril to adults with solid tumors. *Am. J Clin. Onc.*, **12,** 511–518.

STEWART D, MONTPETIT V, MIKHAEL N, DULBERG C, VERMA S, MAROUN J, REDMOND D, MOLEPO M, KEANEY M, DANCEA S AND TRYPHONAS L. (1989*b*). Cisplatin (C) neuropathy (N). *Proc. Am. Assoc. Cancer Res.*, **30,** 246.

STEWART D, GRAHOVAC Z, GIONET L, BENOIT AND HUGENHOLTZ H. (1990*a*). Phase I study of intracarotid carboplatin. *Proc. ASCO*, **9,** 90.

STEWART DJ, VERMA S, MAROUN JA, ROBILLARD L AND EARHART RH. (1990*b*). Phase I study of oral menogaril administered on a once weekly schedule. *Invest. New Drugs*, **8,** 43–52.

STEWART DJ, MOLEPO M, EAPEN L, MONTPETIT V, GOEL R, WONG P, POPOVIC P, TAYLOR K AND RAAPHORST GP. (1994*a*). Cisplatin and radiation in the treatment of tumors of the central nervous system: pharmacological considerations and results of early studies. *Int. J. Radiat. Biol. Phys.*, **28,** 531–542.

STEWART DJ, GREWAAL D, POPOVIC P, MOLEPO JM, SHIRAZI SFH AND GOEL R. (1994*b*). Alteration of cisplatin uptake into lung cancer cell lines. *Proc. Am. Assoc. Cancer Res.*, **35,** 421.

STEWART DJ, DULBERG C, MOLEPO JM, MIKHAEL NZ, MONTPETIT VAJ, REDMOND MD AND GOEL R. (1994*c*). Factors affecting human autopsy kidney cortex and kidney medulla platinum concentrations after cisplatin. *Cancer Chemother. Pharmacol.*, **34,** 14–22.

STEWART PA, HAYAKAWA K, FARRELL CL AND DEL MAESTRO RF. (1987). Quantitative study of microvessel ultrastructure in human peritumoral brain tissue. Evidence for a blood–brain barrier defect. *J. Neurosurg.*, **67,** 697–705.

TATOR CH. (1976). Retention of tritiated methotrexate in a transplantable mouse glioma. *Cancer Res.*, **36,** 3058–3066.

TAYLOR KD, GOEL R, STEWART DJ AND WONG PTT. (1992). Pressure tuning infrared spectroscopic study of cisplatin induced changes in phosphatidylserine model membrane. *Proc. Am. Assoc. Cancer Res.*, **33,** 448.

TAYLOR KD, GOEL R, STEWART DJ AND WONG PTT. (1993). Pressure tuning spectroscopic of cisplatin induced changes in a phosphatidylcholine model membrane. *Proc. Am. Assoc. Cancer Res.*, **34,** 401.

TCHANG S, SCOTT G, TERBRUGGE K, MELANCON D, BELANGER G, MILNER C AND ETHIER R. (1977). Computerized tomography as a possible aid to histological grading of supratentorial gliomas. *J. Neurosurg.*, **46,** 735–739.

TIMMER-BOSSCHA H, HOSPERS GAP, MEIJER C, MULDER NH, MUSKIET FAJ, MARTINI IA, UGES DRA AND DE VRIES EGE. (1989). Influence of docosahexaenoic acid on cisplatin resistance in a human small cell lung carcinoma cell line. *J. Natl Cancer Inst.*, **81,** 1069–1075.

VERMORKEN JB, VAN DER VIJGH WJF, KLEIN I, GALL HE, VAN GROENINGEN CJ, HART GAM AND PINEDO HM. (1986). Pharmacokinetics of free and total platinum species after rapid and prolonged infusions of cisplatin. *Clin. Pharmacol. Ther.*, **39,** 136–144.

WODINSKY I, MERKER PC AND VENFITTI JM. (1977). Responsiveness to chemotherapy of mice with L1210 lymphoid leukemia implanted in various anatomic sites. *J. Natl Cancer Inst.*, **59,** 405.